# Tau pathology and relative cerebral blood flow are independently associated with cognition in Alzheimer’s disease

**DOI:** 10.1007/s00259-020-04831-w

**Published:** 2020-05-27

**Authors:** Denise Visser, Emma E. Wolters, Sander C. J. Verfaillie, Emma M. Coomans, Tessa Timmers, Hayel Tuncel, Juhan Reimand, Ronald Boellaard, Albert D. Windhorst, Philip Scheltens, Wiesje M. van der Flier, Rik Ossenkoppele, Bart N. M. van Berckel

**Affiliations:** 1 Department of Radiology & Nuclear Medicine, Amsterdam Neuroscience, Vrije Universiteit Amsterdam, Amsterdam UMC, Amsterdam, The Netherlands; 2 Alzheimer Center Amsterdam, Department of Neurology, Amsterdam Neuroscience, Vrije Universiteit Amsterdam, Amsterdam UMC, Amsterdam, The Netherlands; 3 Department of Epidemiology and Biostatistics, Vrije Universiteit Amsterdam, Amsterdam UMC, Amsterdam, The Netherlands; 4 Clinical Memory Research Unit, Lund University, Lund, Sweden

**Keywords:** [^18^F]flortaucipir PET, Relative cerebral blood flow, Tau, Cognition, Alzheimer’s disease

## Abstract

**Purpose:**

We aimed to investigate associations between tau pathology and relative cerebral blood flow (rCBF), and their relationship with cognition in Alzheimer’s disease (AD), by using a single dynamic [^18^F]flortaucipir positron emission tomography (PET) scan.

**Methods:**

Seventy-one subjects with AD (66 ± 8 years, mini-mental state examination (MMSE) 23 ± 4) underwent a dynamic 130-min [^18^F]flortaucipir PET scan. Cognitive assessment consisted of composite scores of four cognitive domains. For tau pathology and rCBF, receptor parametric mapping (cerebellar gray matter reference region) was used to create uncorrected and partial volume-corrected parametric images of non-displaceable binding potential (BP_ND_) and *R*_*1*_, respectively. (Voxel-wise) linear regressions were used to investigate associations between BP_ND_ and/or *R*_*1*_ and cognition_._

**Results:**

Higher [^18^F]flortaucipir BP_ND_ was associated with lower *R*_*1*_ in the lateral temporal, parietal and occipital regions. Higher medial temporal BP_ND_ was associated with worse memory, and higher lateral temporal BP_ND_ with worse executive functioning and language. Higher parietal BP_ND_ was associated with worse executive functioning, language and attention, and higher occipital BP_ND_ with lower cognitive scores across all domains. Higher frontal BP_ND_ was associated with worse executive function and attention. For [^18^F]flortaucipir *R*_*1*_, lower values in the lateral temporal and parietal ROIs were associated with worse executive functioning, language and attention, and lower occipital *R*_*1*_ with lower language and attention scores. When [^18^F]flortaucipir BP_ND_ and *R*_*1*_ were modelled simultaneously, associations between lower *R*_*1*_ in the lateral temporal ROI  and worse attention remained, as well as for lower parietal *R*_*1*_ and worse executive functioning and attention.

**Conclusion:**

Tau pathology was associated with locally reduced rCBF. Tau pathology and low rCBF were both independently associated with worse cognitive performance. For tau pathology, these associations spanned widespread neocortex, while for rCBF, independent associations were restricted to lateral temporal and parietal regions and the executive functioning and attention domains. These findings indicate that each biomarker may independently contribute to cognitive impairment in AD.

**Electronic supplementary material:**

The online version of this article (10.1007/s00259-020-04831-w) contains supplementary material, which is available to authorized users.

## Introduction

[^18^F]Flortaucipir is the most widely studied PET tracer to date for detecting AD-specific tau pathology [1]. Most studies with [^18^F]flortaucipir used static scan protocols, which allow semi-quantitative estimates such as the standardized uptake value ratio (SUVR) [1]. Advantages of static over dynamic scanning protocols include the relatively short scan duration and computational simplicity which facilitates clinical applicability [2]. On the other hand, dynamic acquisition allows optimal quantitative accuracy and additionally enables computation of parametric images of tracer delivery, which can be interpreted as a proxy of relative tracer flow or relative cerebral blood flow (rCBF) (i.e. *R*_*1*_) [2–9]. *R*_*1*_ represents the ratio between the rate constant for ligand transfer from blood to tissue (*K*_*1*_) in the tissue of interest and the reference region [4–8], which is strongly correlated with metabolic activity derived from [^18^F]FDG PET [4, 5, 9]. A dynamic [^18^F]flortaucipir PET scan may thus not only provide accurate information on (regional) quantification of tau pathology, but also yields information on rCBF.

Previous studies demonstrated that high levels of regional tau pathology [10–12], as well as low levels of rCBF (as measured with [^18^F]FDG PET or MRI) [9, 13], correlate with cognitive impairment in various domains. However, rCBF has not been investigated yet using [^18^F]flortaucipir *R*_*1*_. Investigating tau pathology and rCBF simultaneously by using dynamic [^18^F]flortaucipir PET might yield valuable information, since both pathophysiological mechanisms may contribute to cognitive impairment in AD.

The aims of this study are to investigate the (regional) association between tau pathology and rCBF, and their (independent) associations with cognitive functioning in patients with AD.

## Methods

### Recruitment of participants

Patients were recruited from the Amsterdam Dementia Cohort of the Alzheimer Center Amsterdam [14]. All subjects underwent a standardized dementia screening, including medical and neurological examination, informant-based history, assessment of vital functions, screening laboratory tests, neuropsychological evaluation, MRI, lumbar puncture and/or amyloid-β positron emission tomography (PET), after which diagnoses were determined in a multidisciplinary consensus meeting [14]. For this study, patients with a diagnosis of Alzheimer’s disease (AD) dementia [15] or mild cognitive impairment (MCI) due to AD [16] were included. For all subjects, AD biomarkers in cerebrospinal fluid (CSF) and/or Aβ PET were abnormal (CSF Aβ42 < 813 pg/mL [17] and/or abnormal Aβ PET (on visual read)). According to the NIA-AA Research Framework [18], all subjects are considered in the AD pathophysiological continuum. Subjects were excluded if they had severe traumatic brain injury, abnormalities on MRI likely to interfere with segmentation of tau PET and participation in drug trial with a tau or Aβ-targeting agent.

All procedures were in accordance with the ethical standards of the Medical Ethics Review Committee of the Amsterdam UMC VU Medical Center and with the 1964 Helsinki Declaration and its later amendments or comparable ethical standards. Informed consent was obtained from all individual participants included in the study.

### Image acquisition

All participants underwent a single dynamic [^18^F]flortaucipir PET scan at the Amsterdam UMC VU Medical Center on an Ingenuity TF PET-CT scanner (Philips Medical Systems, Best, The Netherlands) within 1 year from their neuropsychological examination. [^18^F]Flortaucipir was synthesized at the Amsterdam UMC VU Medical Center, using a protocol described in detail previously [19]. The scan protocol started with a low-dose CT for attenuation correction, followed by a 234 ± 14 MBq [^18^F]flortaucipir bolus injection (injected mass 1 ± 1 μg). Simultaneously with tracer injection, a 60-min dynamic emission scan was initiated. After a 20-min break and following a second low-dose CT for attenuation correction, an additional dynamic emission scan was performed during the interval 80–130 min post-injection. During scanning, head movements were restricted by a head holder with band and head position was regularly checked. PET scans were reconstructed using a matrix size of 128 × 128 × 90 and a final voxel size of 2 × 2 × 2 mm^3^. All standard corrections for dead time, decay, attenuation, randoms and scatter were performed. Both scan sessions were co-registered into a single dataset of 29 frames (1 × 15, 3 × 5, 3 × 10, 4 × 60, 2 × 150, 2 × 300, 4 × 600 and 10 × 300 s), in which the last 10 frames belonged to the second PET session.

In addition, all subjects underwent structural MRI on a 3.0 Tesla (3 T) Philips medical systems’ Ingenuity TF PET-MRI. The protocol included an isotropic structural 3D T1-weighted image using a sagittal turbo gradient-echo sequence (1.00 mm^3^ isotropic voxels, repetition time = 7.9 ms, echo time = 4.5 ms, and flip angle = 8°), and a 3D fluid-attenuated inversion recovery (FLAIR) image (1.04 × 1.04 × 1.12 mm voxels, repetition time = 4800 ms, echo time = 278.8 ms, flip angle 90°).

### PET and MR analyses

Using Vinci software (Max Plank Institute, Cologne, Germany), T1-weighted MR images were co-registered to their individual PET scans in native space. To delineate cortical gray matter regions-of-interest (ROIs) on the co-registered MR images, the Hammers template [20] incorporated in PVElab software was used (which uses the default settings of SPM to define gray matter). To generate voxel-wise parametric images of non-displaceable binding potential (BP_ND_) and *R*_*1*_, receptor parametric mapping (RPM) [21] with cerebellar gray matter as reference region was applied to the dynamic 130 min PET data [22]. Our group previously demonstrated that, when compared to full kinetic modelling, RPM is the most optimal simplified parametric method for [^18^F]flortaucipir [23] with excellent test-retest repeatability [24]. PET images were partial volume-corrected using Van Cittert iterative deconvolution methods (IDM), combined with highly constrained back-projection (HYPR) [25]. A moving frame composite image was used for HYPR to better sustain the temporal information while denoising [26]. Uncorrected data are presented throughout the paper and partial volume-corrected data are presented in the Supplementary material.

For voxel-wise analyses, using Statistical Parametric Mapping (SPM) version 12 software (Wellcome Trust Center for Neuroimaging, University College London, UK), we warped all native space parametric BP_ND_ and *R*_*1*_ images to Montreal Neurological Institute (MNI152) space, by using the transformation matrixes derived from warping the co-registered T1-weighted MRI scans to MNI space. Warped images underwent quality control for transformation errors.

For regional analyses, the following bilateral ROIs were created a priori based on the Hammers atlas [20] (in subject space): medial temporal (hippocampus, parahippocampal and ambient gyri, anterior temporal lobe medial part), lateral temporal (superior temporal gyrus, middle and inferior temporal gyri), parietal (inferolateral remainder of parietal lobe, superior parietal gyrus, gyrus cinguli posterior part), occipital (cuneus, lingual gyrus, lateral remainder of occipital lobe) and frontal (middle frontal gyrus, orbitofrontal gyri, superior frontal gyrus) regions.

As a measure of vascular pathology, white matter hyperintensities (WMHs) were visually rated by an experienced rater on subjects’ FLAIR image using the Fazekas scale, with scores ranging from 0 to 3 [27].

### Cognition

Cognitive domain scores were created by averaging Z-transformed test-scores (based on the current sample) of corresponding tests for memory (Immediate Recall of the Dutch version of the RAVLT, Delayed Recall of the Dutch version of the RAVLT and Visual Association Test-A), executive functioning (Stroop Colour Word test III, Phonemic Verbal Fluency (D-A-T), Digit Span Backwards and Trail Making Test (TMT)-B), language (Category Fluency Animals and Visual Association Test-Naming) and attention (TMT-A, Stroop Colour Word test I and II and the Digit Span Forward) [28]. Tests on which lower scores indicated better performance (TMT-A and -B, Stroop Colour Word test I, II and III) were inverted. Domain scores were only calculated if two or more tests within a domain were available.

### Statistical analyses

To assess the correlations between [^18^F]flortaucipir BP_ND_ and *R*_*1,*_ with age, sex, education and Fazekas score, a correlation matrix was created using Spearman correlations. A *p* value below 0.05 was considered statistically significant.

To examine the regional associations between [^18^F]flortaucipir BP_ND_ and *R*_*1*,_ linear regression analyses, adjusted for age and sex, were performed. To assess the contribution of white matter damage in these associations, analyses were additionally adjusted for Fazekas score.

To assess voxel-wise associations between [^18^F]flortaucipir BP_ND_ or *R*_*1*_ and cognition, voxel-wise regression analyses using SPM12 were performed. Analyses were adjusted for age, sex and education. A *p* value below 0.001 (uncorrected) was considered statistically significant for voxel-wise analyses. Additionally, a more conservative family-wise error (FWE) correction at *p* < 0.05 was applied.

To investigate regional associations between [^18^F]flortaucipir BP_ND_ or *R*_*1*_ and cognition (dependent variables), linear regression analyses, adjusted for age, sex and education (model 1), were used. Subsequently, we entered [^18^F]flortaucipir BP_ND_ and *R*_*1*_ simultaneously in the model to assess their independent associations with cognition (model 2).

For all regional analyses, we report the level of significance both with and without correction for multiple comparisons using the Benjamini-Hochberg procedure with a false discovery rate (FDR) Q value of 5%. A *p* value below 0.05 was considered statistically significant. All regional and voxel-wise analyses were repeated with partial volume-corrected data.

## Results

### Participants

A total of 71 subjects (MCI due to AD: *n* = 10, and AD dementia: *n* = 61) with a mean age of 66 ± 8 years and MMSE score of 23 ± 4 were included (Table 1). By study design, all subjects had abnormal amyloid biomarkers. [^18^F]Flortaucipir BP_ND_ values were highest in parietal (0.55 ± 0.43) regions and *R*_*1*_ values were lowest in medial temporal regions (0.68 ± 0.06) (Table 1). [^18^F]Flortaucipir BP_ND_ and/or *R*_*1*_ showed statistically significant correlations with age and education (Table 2), but not with sex.

**Table 1:** Overview of demographics, [^18^F]flortaucipir BP_ND_ and *R*_*1*_

	*N* = 71
Diagnosis	
MCI due to AD (*n*)	10
AD dementia (*n*)	61
Age (years)	66 (8)
Sex (female/male)	36/35
Education (Dutch Verhage scale)	6 [3–7]
Fazekas score	1 [0–3]
MMSE	23 (4)
[^18^F]flortaucipir BP_ND_	
Medial temporal	0.25 ± 0.15 [− 0.11–0.59]
Lateral temporal	0.48 ± 0.30 [− 0.12–1.29]
Parietal	0.55 ± 0.43 [− 0.16–1.83]
Occipital	0.45 ± 0.40 [− 0.05–1.82]
Frontal	0.26 ± 0.27 [− 0.22–0.94]
[^18^F]flortaucipir *R*_*1*_	
Medial temporal	0.68 ± 0.06 [0.57–0.86]
Lateral temporal	0.86 ± 0.08 [0.70–1.13]
Parietal	0.87 ± 0.11 [0.61–1.30]
Occipital	0.98 ± 0.10 [0.74–1.34]
Frontal	0.88 ± 0.07 [0.74–1.11]

Mean (SD) are reported for all variables, except for diagnosis (*n*), sex (*n*_female_/*n*_male_) and education and Fazekas score (median [range]). For [^18^F]flortaucipir BP_ND_ and *R*_*1*,_ the range is additionally provided. Parametric [^18^F]flortaucipir images were not partial volume-corrected. *MCI* mild cognitive impairment, *AD* Alzheimer’s disease, *MMSE* Mini-Mental State Examination, *BP*_*ND*_ non-displaceable binding potential

**Table 2:** Correlation matrix (Spearman’s rho) for BP_ND_ and *R*_*1*_ and (possible) covariates age, sex, education and Fazekas score

Correlation matrix (Spearman’s rho)	Age	Sex	Education	Fazekas score	BP_ND_ medial temporal	BP_ND_ lateral temporal	BP_ND_ parietal	BP_ND_ occipital	BP_ND_ frontal	*R*_*1*_ medial temporal	*R*_*1*_ lateral temporal	*R*_*1*_ parietal	*R*_*1*_ occipital	*R*_*1*_ frontal
Age	1.00													
Sex	− 0.09	1.00												
Education	− 0.05	− 0.03	1.00											
Fazekas score	0.48^‡§^	− 0.15	− 0.07	1.00										
BP_ND_ medial temporal	0.02	− 0.04	− 0.25*	0.14	1.00									
BP_ND_ lateral temporal	− 0.31^†§^	− 0.05	− 0.17	0.01	0.68^‡§^	1.00								
BP_ND_ parietal	− 0.64^‡§^	0.04	0.00	− 0.19	0.32^†§^	0.73^‡§^	1.00							
BP_ND_ occipital	− 0.46^‡§^	− 0.01	− 0.01	− 0.19	0.32^†§^	0.62^‡§^	0.84^‡§^	1.00						
BP_ND_ frontal	− 0.47^‡§^	− 0.01	− 0.02	− 0.03	0.49^‡§^	0.77^‡§^	0.82^‡§^	0.57^‡§^	1.00					
*R*_*1*_ medial temporal	− 0.38^‡§^	0.03	− 0.01	− 0.21	− 0.10	− 0.04	0.29*^§^	0.14	0.20	1.00				
*R*_*1*_ lateral temporal	− 0.11	0.18	0.01	− 0.20	− 0.19	− 0.30*^§^	− 0.04	− 0.03	− 0.12	0.67^‡§^	1.00			
*R*_*1*_ parietal	0.17	0.09	0.02	− 0.14	− 0.10	− 0.32^†§^	− 0.37^†§^	− 0.40^‡§^	− 0.27*^§^	0.21	0.68^‡§^	1.00		
*R*_*1*_ occipital	0.08	− 0.01	− 0.03	− 0.15	− 0.01	− 0.13	− 0.20	− 0.42^‡§^	0.02	0.24*	0.45^‡§^	0.77^‡§^	1.00	
*R*_*1*_ frontal	− 0.11	0.19	− 0.03	− 0.23	− 0.18	− 0.16	0.08	0.13	− 0.06	0.64^‡§^	0.81^‡§^	0.52^‡§^	0.22	1.00

*p < 0.05, ^†^*p* < 0.01, ^‡^*p* < 0.001, ^§^*p*_FDR_ < 0.05

### Associations between [^18^F]flortaucipir BP_ND_ and *R*_*1*_

**Fig. 1:**
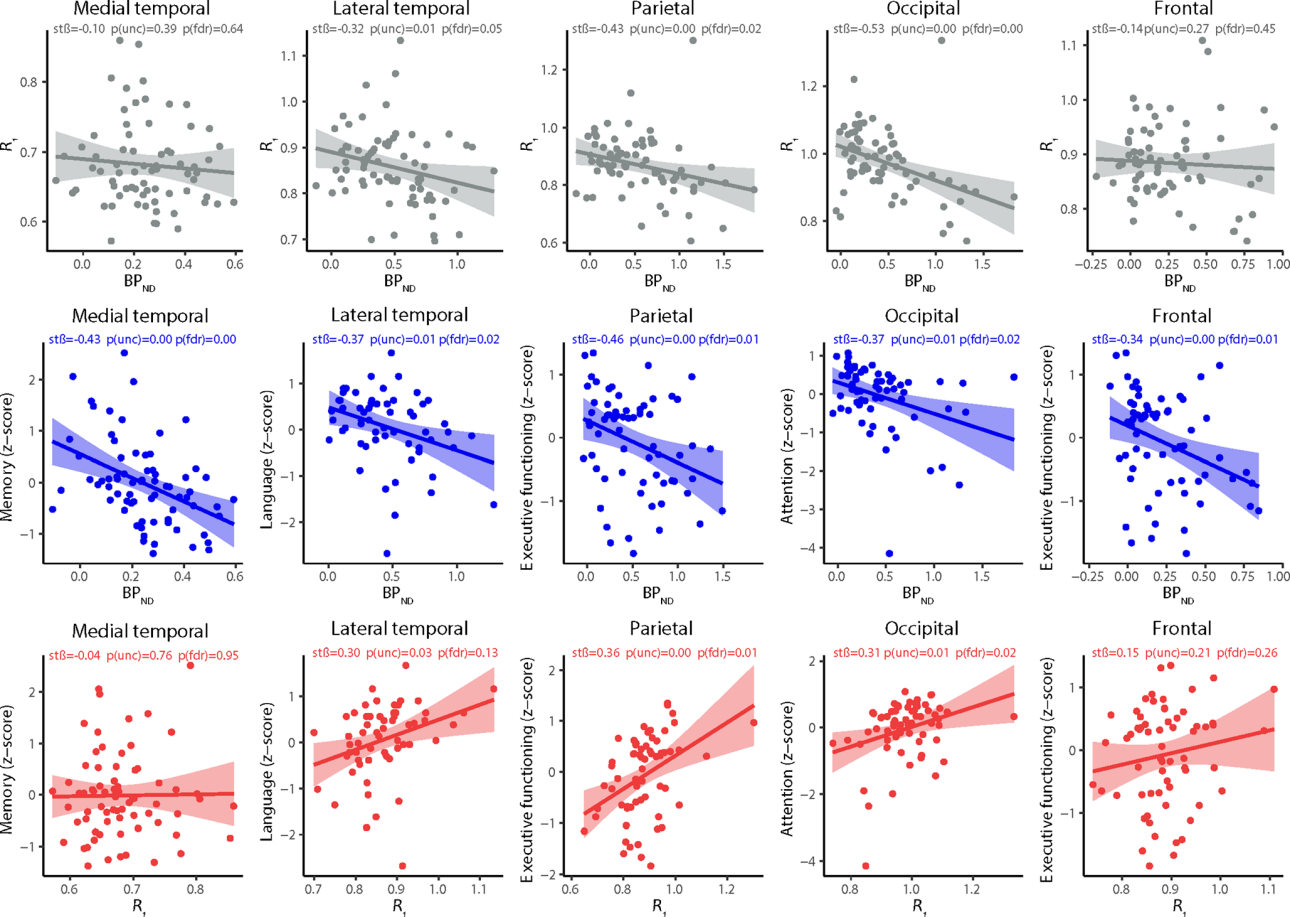
Selection of scatterplots between [^18^F]flortaucipir BP_ND_ and/or *R*_*1*_ and/or cognition. *BP*_*ND*_ non-displaceable binding potential, *stβ* standardized β, *p(unc)* uncorrected *p* value, *p(fdr)* *p* value corrected for multiple comparisons using the Benjamini-Hochberg procedure with a false discovery rate (FDR) Q value of 5%. A *p* value below 0.05 was considered statistically significant

Higher [^18^F]flortaucipir BP_ND_ was associated with lower *R*_*1*_ within the lateral temporal (stβ − 0.32 [95%CI − 0.56 to − 0.08]), parietal (− 0.43 [− 0.72 to − 0.14]) and occipital (− 0.53 [− 0.78 to − 0.29]) ROI (Table 3). Higher BP_ND_ in the occipital ROI was also associated with lower *R*_*1*_ in the parietal ROI (− 0.38 [− 0.64 to − 0.12]). All associations remained significant after FDR correction (Table 3). Figure 1 shows a selection of scatterplots for these associations. Addition of Fazekas scores to the model did not notably change the results (Supplementary Table 1).

**Table 3:** Regional association between [^18^F]flortaucipir BP_ND_ (rows) and *R*_*1*_ (columns)

[^18^F]flortaucipir *R*_*1*_	Medial temporal	Lateral temporal	Parietal	Occipital	Frontal
[^18^F]flortaucipir BP_ND_					
Medial temporal	− 0.10 [− 0.32–0.13]	− 0.21 [− 0.44–0.02]	− 0.11 [− 0.35–0.13]	0.03 [− 0.21–0.27]	− 0.18 [− 0.41–0.05]
Lateral temporal	− 0.15 [− 0.38–0.08]	− 0.32*^‡^ [− 0.56– − 0.08]	− 0.24 [− 0.49–0.01]	− 0.10 [− 0.35–0.16]	− 0.19 [− 0.43–0.06]
Parietal	0.10 [− 0.18–0.38]	− 0.14 [− 0.44–0.16]	− 0.43*^‡^ [− 0.72– − 0.14]	− 0.29 [− 0.59–0.00]	0.04 [− 0.26–0.33]
Occipital	0.02 [− 0.24–0.27]	− 0.07 [− 0.34–0.20]	− 0.38*^‡^ [− 0.64– − 0.12]	− 0.53^†‡^ [− 0.78– − 0.29]	0.13 [− 0.13–0.40]
Frontal	0.12 [− 0.12–0.36]	− 0.18 [− 0.43–0.08]	− 0.23 [− 0.49–0.03]	0.09 [− 0.18–0.35]	− 0.14 [− 0.40–0.11]

Model is adjusted for age and sex. Standardized β’s with 95% confidence intervals are reported. *BP*_*ND*_ non-displaceable binding potential. *p < 0.01, ^†^*p* < 0.001, ^‡^*p*_FDR_ < 0.05

### Voxel-wise associations with cognition

**Fig. 2:**
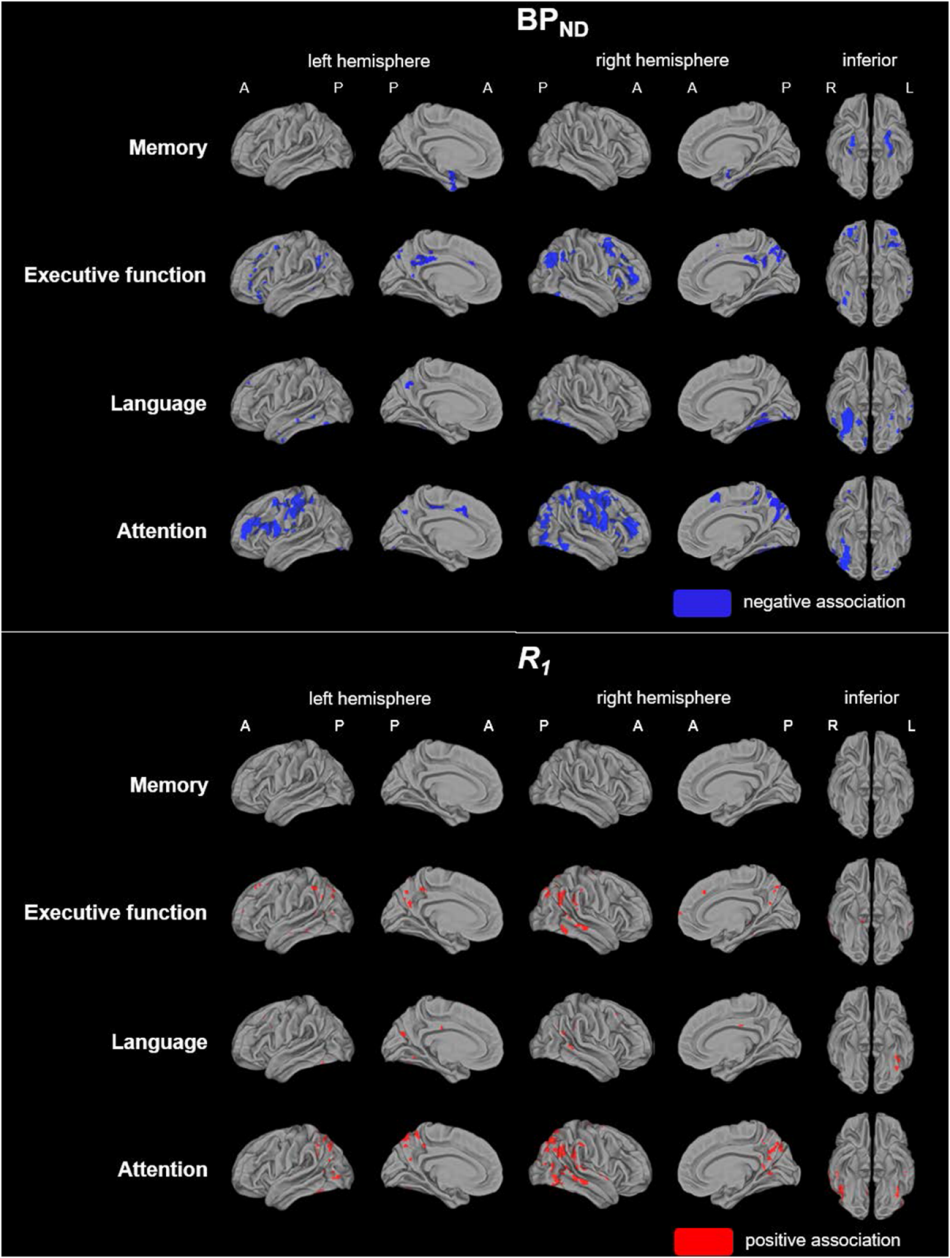
Voxel-wise associations between [^18^F]flortaucipir BP_ND_ or *R*_*1*_ and cognition. Voxel-wise regression analyses were performed, adjusted for age, sex and education. Voxels with a significant (*p* < 0.001, uncorrected) association are displayed. *BP*_*ND*_ non-displaceable binding potential, *A* anterior, *P* posterior, *R* right, *L* left

Voxel-wise analyses (model 1) revealed that, in general, higher [^18^F]flortaucipir BP_ND_ was associated with worse cognition (Fig. 2). More specifically, higher medial temporal BP_ND_ associated with worse memory performance, and higher (orbito-)frontoparietal BP_ND_ with worse scores on executive functioning (Fig. 2). Higher inferior temporal BP_ND_ associated with worse language performance and higher (middle-)frontoparietal and occipital BP_ND_ with worse attention scores (Fig. 2). After FWE correction, sparse associations with higher BP_ND_ in the medial temporal regions and worse memory performance remained, as well as higher BP_ND_ in the temporal (fusiform cortex) regions and worse language scores (data not shown). Associations between higher BP_ND_ in the parietal and frontal regions and worse attention also survived FWE correction (data not shown).

Overall, lower *R*_*1*_ associated with worse cognition (Fig. 2). In more detail, lower fronto-temporoparietal *R*_*1*_ associated with worse scores on executive functioning and to a sparser extent with worse language performance (Fig. 2). Lower temporoparietal *R*_*1*_ associated with worse attention scores (Fig. 2). None of the associations survived FWE correction (data not shown).

### Regional associations with cognition

**Fig. 3:**
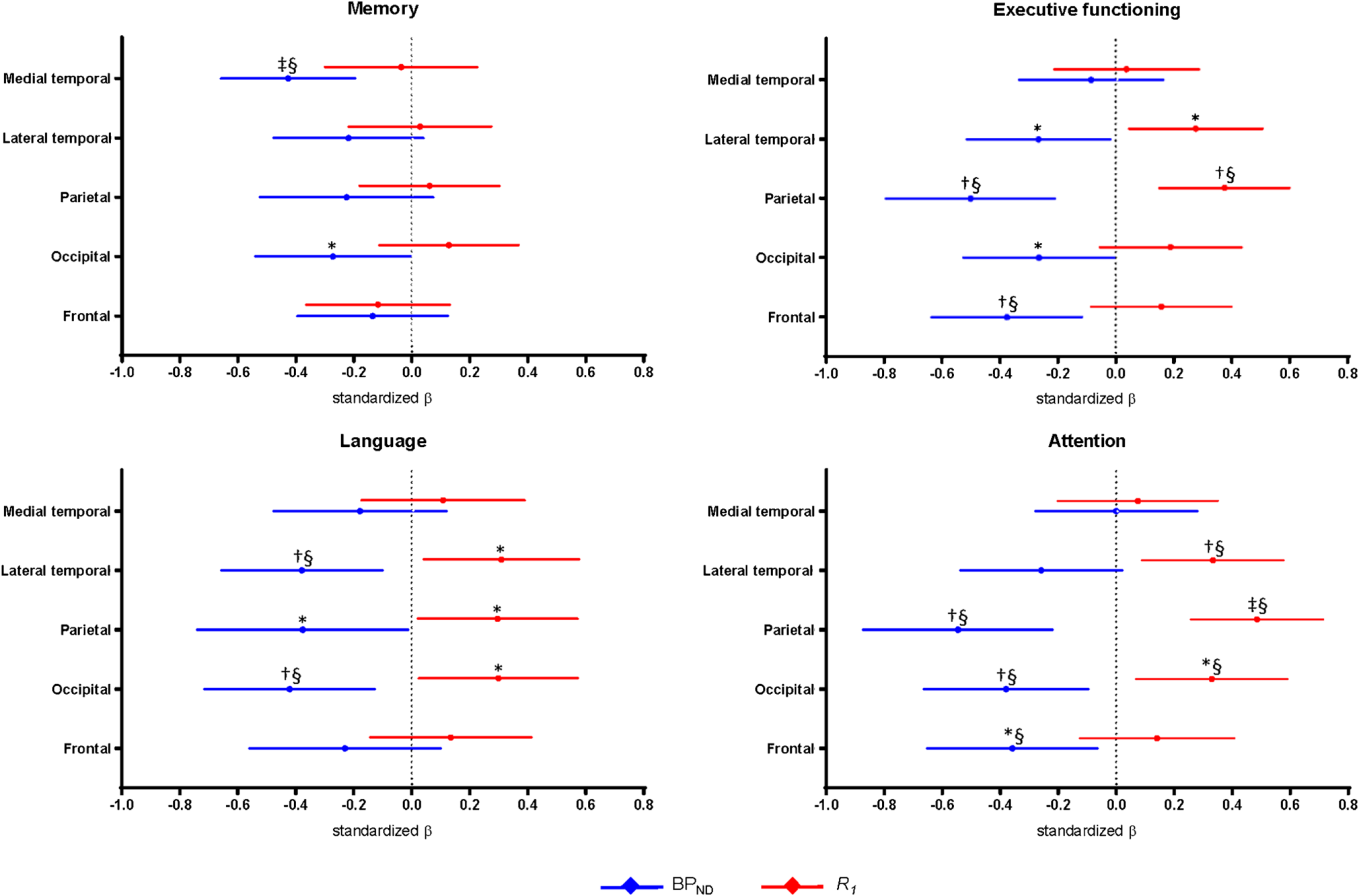
Regional associations between [^18^F]flortaucipir BP_ND_ or *R*_*1*_ and cognition. [^18^F]Flortaucipir BP_ND_ and *R*_*1*_ were included in the model separately (model 1). Displayed are regression estimates (standardized β’s) with 95% confidence intervals. All analyses are adjusted for age, sex and education. *BP*_*ND*_ non-displaceable binding potential. *p < 0.05, ^†^*p* < 0.01, ^‡^*p* < 0.001, ^§^*p*_FDR_ < 0.05

Regional linear regression analyses (model 1) revealed that higher medial temporal BP_ND_ was associated with worse memory performance (− 0.43 [− 0.66 to − 0.20]), higher lateral temporal BP_ND_ with worse scores on executive functioning (− 0.26 [− 0.52 to − 0.02]) and language (− 0.37 [− 0.66 to − 0.11]), and higher parietal BP_ND_ with worse executive functioning (− 0.46 [− 0.81 to − 0.23]), language (− 0.34 [− 0.76 to − 0.03]) and attention (− 0.50 [− 0.89 to − 0.25]) (Table 4; Fig. 3). Higher BP_ND_ in the occipital ROI was associated with worse memory (− 0.27 [− 0.54 to − 0.00]), executive functioning (− 0.26 [− 0.53 to − 0.01]), language (− 0.40 [− 0.73 to − 0.14]) and attention (− 0.37 [− 0.67 to − 0.10]) performance, and higher BP_ND_ in the frontal ROI with worse executive functioning (− 0.34 [− 0.65 to − 0.13]) and attention (− 0.33 [− 0.67 to − 0.08]). After FDR correction, the majority of significant associations remained (Table 4; Fig. 3).

**Table 4:** Regional associations between [^18^F]flortaucipir BP_ND_ and *R*_*1*_ and cognition

		Memory (*n* = 71)	Executive functioning (*n* = 64)	Language (*n* = 59)	Attention (*n* = 64)
Model 1					
Medial temporal	BP_ND_	− 0.43^‡§^ [− 0.66– − 0.20]	− 0.08 [− 0.34–0.16]	− 0.17 [− 0.48–0.12]	0.00 [− 0.28–0.28]
	*R*_*1*_	− 0.04 [− 0.30–0.22]	0.03 [− 0.22–0.28]	0.10 [− 0.18–0.38]	0.07 [− 0.21–0.35]
Lateral temporal	BP_ND_	− 0.22 [− 0.48–0.04]	− 0.26* [− 0.52– − 0.02]	− 0.37^†§^ [− 0.66– − 0.11]	− 0.25 [− 0.54–0.02]
	*R*_*1*_	0.03 [− 0.22–0.27]	0.27* [0.04–0.50]	0.30* [0.04–0.57]	0.33^†§^ [0.08–0.57]
Parietal	BP_ND_	− 0.23 [− 0.52–0.07]	− 0.46^†§^ [− 0.81– − 0.23]	− 0.34* [− 0.76– − 0.03]	− 0.50^†§^ [− 0.89– − 0.25]
	*R*_*1*_	0.06 [− 0.18–0.30]	0.36^†§^ [0.15–0.60]	0.28* [0.02–0.57]	0.48^‡§^ [0.26–0.71]
Occipital	BP_ND_	− 0.27* [− 0.54– − 0.00]	− 0.26* [− 0.53– − 0.01]	− 0.40^†§^ [− 0.73– − 0.14]	− 0.37^†§^ [− 0.67– − 0.10]
	*R*_*1*_	0.13 [− 0.12–0.37]	0.17 [− 0.06–0.43]	0.28* [0.03–0.57]	0.31*^§^ [0.07–0.59]
Frontal	BP_ND_	− 0.14 [− 0.40–0.12]	− 0.34^†§^ [− 0.65– − 0.13]	− 0.21 [− 0.57–0.09]	− 0.33*^§^ [− 0.67– − 0.08]
	*R*_*1*_	− 0.12 [− 0.37–0.13]	0.15 [− 0.09–0.40]	0.13 [− 0.15–0.41]	0.13 [− 0.13–0.40]
Model 2					
Medial temporal	BP_ND_	− 0.44^‡§^ [− 0.67– − 0.20]	− 0.08 [− 0.34–0.17]	− 0.17 [− 0.48–0.12]	0.01 [− 0.28–0.29]
	*R*_*1*_	− 0.09 [− 0.33–0.15]	0.03 [− 0.23–0.28]	0.10 [0.19–0.38]	0.07 [− 0.21–0.35]
Lateral temporal	BP_ND_	− 0.23 [− 0.51–0.04]	− 0.19 [− 0.46–0.05]	− 0.31* [− 0.60– − 0.04]	− 0.16 [− 0.45–0.11]
	*R*_*1*_	− 0.05 [− 0.30–0.21]	0.21 [− 0.02–0.46]	0.22 [− 0.04–0.49]	0.28* [0.03–0.54]
Parietal	BP_ND_	− 0.23 [− 0.55–0.09]	− 0.36^†§^ [− 0.70– − 0.11]	− 0.27 [− 0.68–0.05]	− 0.36* [− 0.72– − 0.10]
	*R*_*1*_	− 0.00 [− 0.26–0.25]	0.27* [0.06–0.50]	0.22 [− 0.05–0.52]	0.39^†§^ [0.17–0.62]
Occipital	BP_ND_	− 0.26 [− 0.57–0.04]	− 0.21 [− 0.52–0.07]	− 0.33* [− 0.68– − 0.03]	− 0.27 [− 0.59–0.03]
	*R*_*1*_	0.02 [− 0.25–0.28]	0.09 [− 0.17–0.37]	0.15 [− 0.13–0.46]	0.20 [− 0.07–0.50]
Frontal	BP_ND_	− 0.16 [− 0.42–0.11]	− 0.33^†§^ [− 0.64– − 0.11]	− 0.20 [− 0.56–0.10]	− 0.31*^§^ [− 0.66– − 0.06]
	*R*_*1*_	− 0.14 [− 0.39–0.11]	0.11 [− 0.12–0.34]	0.11 [− 0.16–0.39]	0.09 [− 0.17–0.35]

Models are adjusted for age, sex and education. [^18^F]Flortaucipir BP_ND_ and *R*_*1*_ were included in the model separately (model 1) and simultaneously (model 2). Standardized β’s with 95% confidence intervals are reported. *BP*_*ND*_ non-displaceable binding potential. *p < 0.05, ^†^*p* < 0.01, ^‡^*p* < 0.001, ^§^*p*_FDR_ < 0.05

Lower lateral temporal and parietal *R*_*1*_ was associated with lower scores on executive functioning (0.27 [0.04 to 0.50]; 0.36 [0.15 to 0.60]), language (0.30 [0.04 to  0.57]; 0.28 [0.02 to 0.57]) and attention (0.33 [0.08 to 0.57]; 0.48 [0.26 to 0.71]) (Table 4; Fig. 3). Lower *R*_*1*_ in the occipital ROI was associated with worse language (0.28 [0.03 to 0.57]) and attention (0.31 [0.07 to 0.59]) performance. By applying the FDR correction, the significant associations between lower *R*_*1*_ in the parietal ROI and worse executive functioning remained, as well as the significant associations between lower *R*_*1*_ in the lateral temporal, parietal and occipital ROI and worse attention scores (Table 4; Fig. 3). Scatterplots for a selection of these associations are presented in Fig. 1.

Finally, to examine the independent effects of tau pathology and rCBF on cognitive functioning, linear regression analyses including both [^18^F]flortaucipir BP_ND_ and *R*_*1*_ were performed (model 2) (Table 4). Results revealed that higher medial temporal BP_ND_ was independently associated with worse memory (− 0.44 [− 0.67 to − 0.20]), higher lateral temporal BP_ND_ with worse language (− 0.31 [− 0.60 to − 0.04]) performance, and higher parietal BP_ND_ with worse scores on executive functioning (− 0.36 [− 0.70 to − 0.11]) and attention (− 0.36 [− 0.72 to − 0.10]). Higher occipital BP_ND_ was independently associated with worse language (− 0.33 [− 0.68 to − 0.03]) and higher frontal BP_ND_ with lower scores on executive functioning (− 0.33 [− 0.64 to − 0.11]) and attention (− 0.31 [− 0.66 to − 0.06]). Most significant associations survived FDR correction (Table 4). For [^18^F]flortaucipir *R*_*1*_, lower values in the lateral temporal ROI were independently associated with worse attention (0.28 [0.03 to 0.54]), while low *R*_*1*_ values in the parietal ROI were with lower scores on executive functioning (0.27 [0.06 to 0.50]) and attention (0.39 [0.17 to 0.62]). After FDR correction, the association between lower *R*_*1*_ in the parietal ROI and worse attention remained (Table 4).

### Additional analyses

Overall, partial volume-corrected data yielded slightly higher values for both [^18^F]flortaucipir BP_ND_ and *R*_*1*_ (Supplementary Table 2), but results from regression analyses remained essentially comparable (Supplementary Tables 3 & 4; Supplementary FIGs. 1 & 2).

## Discussion

The present study used a single dynamic [^18^F]flortaucipir PET scan to examine the relationship between tau pathology, rCBF and cognition in AD. The main finding is that high levels of tau pathology and low levels of rCBF were independently associated with worse cognitive performance across various domains.

### Tau pathology and rCBF are independently associated with cognition in AD

An important finding in the present study is that tau pathology and rCBF, at least in part, independently contribute to cognitive deficits in AD. A previous study demonstrated that tau pathology was also independently associated with specific cognitive impairment in AD in the context of neurodegeneration [29]. This leads to the notion that tau pathology may impact cognitive performance directly, but also indirectly through a variety of mechanisms [29]. One such mechanisms might be rCBF, since some of the associations found between [^18^F]flortaucipir BP_ND_ and cognition in the present study disappeared when *R*_*1*_ was included in the model simultaneously. Other factors possibly explaining the tau pathology-independent associations between rCBF and cognition in AD might be the presence of other down- or upstream pathological factors like tau-independent atrophy, vascular pathology or other proteinopathies. Vascular damage for example might lead to impaired rCBF, possibly causing an increase in amyloid-β accumulation, which in turn can lead to inflammation and neuronal dysfunction, leading to cognitive deficits [30]. Further research is required, however, to gain knowledge about the mechanisms explaining the tau-independent relationships between rCBF and cognition in AD.

### Associations between tau pathology, rCBF and cognition

Strong (regional) associations between tau pathology and cognitive deficits in AD have been established by multiple (imaging) studies [10–12, 31], and results of the present study are generally in line with previous findings. As expected, tau pathology in the medial temporal regions showed strong associations with memory, while tau pathology in temporoparietal regions was associated with language. High levels of tau pathology in frontal regions were associated with more anteriorly based cognitive functions, like executive function and attention.

Although the association between CBF and cognition in AD has not been studied using this [^18^F]flortaucipir PET approach before, other studies investigated these associations by using [^18^F]FDG PET or MRI techniques (such as arterial spin labeling (ASL)) to measure (proxies of) CBF [13, 32]. These studies demonstrated that in AD, reduced CBF is generally associated with worse global cognition [13, 32] and not with domain-specific cognitive impairments (memory, executive function, language, attention and visuospatial functioning) [13]. Nonetheless, it was also demonstrated that most associations with cognition were found for low CBF in parietal and occipital regions, while least associations were found for temporal and frontal CBF [13]. This is in line with our results, although we also found multiple associations between cognition and low CBF in lateral temporal regions. This might be explained by the fact that the former study used a ROI covering the entire temporal cortex and did not differentiate between medial and lateral temporal regions. Both studies used subjects from the Amsterdam Dementia Cohort and used a similar approach to assess cognition, but another striking similarity between the former and present study is the range of regression coefficients for significant associations between CBF and cognition. Standardized regression coefficients ranged from 0.22 till 0.42 across the cognitive domains in the former study [13], and ranged from 0.27 till 0.48 in the present study, indicating comparable effect sizes.

### Regional association between tau pathology and low rCBF

Relative CBF is tightly correlated with measures of metabolic activity such as [^18^F]FDG PET [4, 5, 9]. Earlier studies investigating [^18^F]flortaucipir and [^18^F]FDG PET in AD found considerable overlap between higher levels of tau tracer uptake and lower levels of metabolic activity [11, 33], with moderate correlation coefficients across 30 predefined brain regions. The present study used *R*_*1*_ as proxy for rCBF, and in line with the previously described study [33], we also found spatial overlap between high levels of tau pathology and low rCBF, with comparable standardized regression coefficients. The overlap of high levels of tau pathology and low levels of rCBF was in both studies not completely uniform across all brain regions, suggesting that both measures represent complementary aspects of AD pathology [33]. A potential explanation might be that tau pathology may develop prior to or even (partially) drive impaired metabolic activity or CBF, creating a time-lag between both pathological mechanism leading to topographical differences [34]. Alternatively, other pathological processes besides tau pathology may contribute to impaired metabolic activity or CBF, such as other proteinopathies. Vascular pathology has been linked to AD [35] and might have an impact on for example rCBF. However, in our study, the influence of vascular pathology showed to be negligible, since no correlation between Fazekas score and tau pathology or rCBF was found, and addition of Fazekas score to the regression model assessing the association between tau pathology and rCBF did not notably change results.

### Strengths and limitations

This study has several strengths, including the use of [^18^F]flortaucipir *R*_*1*_ as a measure of rCBF, since this tracer has not been used in this context before, while [^18^F]flortaucipir currently is the most widely used tracer for tau pathology in AD. Another strength is that both measures were derived from a single dynamic [^18^F]flortaucipir PET scan, thereby circumventing the need for a dual-tracer study and avoiding bias caused by time-lags between measures of tau pathology and rCBF. Furthermore, analyses were repeated with partial volume-corrected data, and results remained essentially comparable; hence, we feel that our findings are not biased by atrophy to a large extent.

This study also has some limitations. The AD patients in this study were relatively young, which might hamper generalizability of results to older patient populations. Also, because our sample included only ten ‘MCI due to AD’ patients, further research is needed to elucidate potential differences in the BP_ND_-*R*_*1*_ relationship between diagnostic groups. Furthermore, it might be difficult to draw firm conclusions about the performance of [^18^F]flortaucipir *R*_*1*_ compared to other measures of CBF due to the lack of a golden standard for measuring CBF. At last, this study has a cross-sectional design, which excluded the possibility to investigate whether the associations found between tau pathology, rCBF and cognition in AD represent causality. Therefore, longitudinal designs are required.

## Conclusion

This study demonstrates that tau pathology and rCBF derived from a single dynamic [^18^F]flortaucipir PET scan are associated in a region-specific matter, with high levels of tau pathology being generally present in areas with low levels of rCBF. Lower cognitive scores are associated with higher levels of tau pathology and lower levels of rCBF. A substantial amount of these associations remained present when correcting for the other PET measure, indicating that tau pathology and rCBF (at least in part) independently contribute to cognitive deficits in AD. Besides, this study indicates that the use of dynamic [^18^F]flortaucipir PET might sometimes be preferable, since accurate quantification of tau pathology and an additional functional measure like rCBF can be derived from a single scan.

## Electronic supplementary material

Supplementary Table 1 (DOCX 13.3 kb).

Supplementary Table 2 (DOCX 12.4 kb).

Supplementary Table 3 (DOCX 13.2 kb).

Supplementary Table 4 (DOCX 14.8 kb).

Supplementary Figure 1 (PNG 2485 kb).

High Resolution Image (TIFF 863 kb).

Supplementary Figure 2 (PNG 4979 kb).

High Resolution Image (TIFF 241 kb).
