# Supplementary material for: Tau pathology and relative cerebral blood flow are independently associated with cognition in Alzheimer’s disease
Source: Eur J Nucl Med Mol Imaging. 2020 May 27;47(13):3165–75. doi: 10.1007/s00259-020-04831-w (PMC7680306; doi:10.1007/s00259-020-04831-w)
Supplement: Supplementary file 1 — (DOCX 13.3 kb). [file 259_2020_4831_MOESM1_ESM.docx]

**Supplementary TABLE 1** **Regional association between [^18^F]flortaucipir BP_ND_ (rows) and *R_1_* (columns), corrected for Fazekas score.**

| [^18^F]flortaucipir *R_1_* | Medial temporal | Lateral temporal | Parietal | Occipital | Frontal |
| --- | --- | --- | --- | --- | --- |
| [^18^F]flortaucipir BP_ND_  Medial temporal  Lateral temporal  Parietal  Occipital  Frontal | -0.10 [-0.32 – 0.13]  -0.15 [-0.39 – 0.09]  0.11 [-0.18 – 0.39]  0.02 [-0.24 – 0.27]  0.13 [-0.11 – 0.38] | -0.20 [-0.44 – 0.04]  **-0.31* [-0.56** − **-0.07]**  -0.13 [-0.43 – 0.17]  -0.07 [-0.34 – 0.21]  -0.16 [-0.42 – 0.10] | -0.09 [-0.34 – 0.15]  -0.23 [-0.48 – 0.03]  **-0.42^†§^ [-0.71** − **-0.13]**  **-0.38^†§^ [-0.64** − **-0.12]**  -0.22 [-0.48 – 0.05] | 0.06 [-0.19 – 0.30]  -0.07 [-0.33 – 0.19]  -0.28 [-0.57 – 0.02]  **-0.53^‡§^ [-0.78** − **-0.29]**  0.11 [-0.15 – 0.38] | -0.16 [-0.40 – 0.08]  -0.17 [-0.42 - 0.08]  0.05 [-0.24 – 0.35]  0.14 [-0.13 – 0.40]  -0.12 [-0.38 - 0.13] |

Model is adjusted for age, sex and Fazekas score. Standardized β’s with 95% confidence intervals are reported. BP_ND_ = non-displaceable binding potential. *p<0.05, ^†^p<0.01, ^‡^p<0.001, ^§^p_FDR_<0.05.
