# Supplementary material for: Tau pathology and relative cerebral blood flow are independently associated with cognition in Alzheimer’s disease
Source: Eur J Nucl Med Mol Imaging. 2020 May 27;47(13):3165–75. doi: 10.1007/s00259-020-04831-w (PMC7680306; doi:10.1007/s00259-020-04831-w)
Supplement: Supplementary file 2 — (DOCX 12.4 kb). [file 259_2020_4831_MOESM2_ESM.docx]

**Supplementary TABLE 2 Overview of partial volume corrected [^18^F]flortaucipir BP_ND_ and *R_1_*.**

|  | N=71 |
| --- | --- |
| [^18^F]flortaucipir BP_ND_  Medial temporal  Lateral temporal  Parietal  Occipital  Frontal | 0.28 (0.18)  0.54 (0.35)  0.69 (0.52)  0.52 (0.46)  0.35 (0.32) |
| [^18^F]flortaucipir *R_1_*  Medial temporal  Lateral temporal  Parietal  Occipital  Frontal | 0.69 (0.07)  0.88 (0.09)  0.94 (0.13)  1.02 (0.12)  0.95 (0.09) |

Mean (SD) are reported for all variables. Parametric [^18^F]flortaucipir images were partial volume corrected. BP_ND_ = non-displaceable binding potential.
