# Supplementary material for: Tau pathology and relative cerebral blood flow are independently associated with cognition in Alzheimer’s disease
Source: Eur J Nucl Med Mol Imaging. 2020 May 27;47(13):3165–75. doi: 10.1007/s00259-020-04831-w (PMC7680306; doi:10.1007/s00259-020-04831-w)
Supplement: Supplementary file 3 — (DOCX 13.2 kb). [file 259_2020_4831_MOESM3_ESM.docx]

**Supplementary TABLE 3** **Regional association between partial volume corrected [^18^F]flortaucipir BP_ND_ (rows) and *R_1_* (columns).**

| [^18^F]flortaucipir *R_1_* | Medial temporal | Lateral temporal | Parietal | Occipital | Frontal |
| --- | --- | --- | --- | --- | --- |
| [^18^F]flortaucipir BP_ND_  Medial temporal  Lateral temporal  Parietal  Occipital  Frontal | -0.07 [-0.29 – 0.15]  -0.12 [-0.35 – 0.10]  0.03 [-0.23 – 0.30]  0.00 [-0.25 – 0.25]  0.10 [-0.13 – 0.34] | -0.21 [-0.44 – 0.02]  -0.24 [-0.48 – 0.00]  -0.17 [-0.33 – 0.20]  -0.07 [-0.33 – 0.20]  -0.15 [-0.40 – 0.10] | -0.22 [-0.46 – 0.02]  **-0.26* [-0.51** − **-0.02]**  **-0.43^†§^ [-0.71** − **-0.16]**  **-0.33*^§^ [-0.59** − **-0.07]**  -0.25 [-0.50 - -0.01] | -0.12 [-0.36 - 0.12]  -0.18 [-0.43 – 0.07]  **-0.38^†§^ [-0.66** − **-0.10]**  **-0.48^‡§^ [-0.73** − **-0.24]**  -0.04 [-0.30 – 0.22] | -0.11 [-0.34 – 0.13]  -0.07 [-0.31 – 0.17]  0.01 [-0.27 – 0.29]  0.09 [-0.17 – 0.35]  -0.04 [-0.29 – 0.21] |

Model is adjusted for age and sex. Standardized β’s with 95% confidence intervals are reported. Parametric [^18^F]flortaucipir images were partial volume corrected. BP_ND_ = non-displaceable binding potential. *p<0.05, ^†^p<0.01, ^‡^p<0.001, ^§^p_FDR_<0.05.
