# Supplementary material for: Tau pathology and relative cerebral blood flow are independently associated with cognition in Alzheimer’s disease
Source: Eur J Nucl Med Mol Imaging. 2020 May 27;47(13):3165–75. doi: 10.1007/s00259-020-04831-w (PMC7680306; doi:10.1007/s00259-020-04831-w)
Supplement: Supplementary file 4 — (DOCX 14.8 kb). [file 259_2020_4831_MOESM4_ESM.docx]

**Supplementary TABLE 4** **Regional associations between partial volume corrected [^18^F]flortaucipir BP_ND_ and *R_1_*  and cognition.**

|  |  | **Memory (n=71)** | **Executive functioning (n=64)** | **Language (n=59)** | **Attention (n=64)** |
| --- | --- | --- | --- | --- | --- |
| ***Model 1*** |  |  |  |  |  |
| **Medial temporal** | **BP_ND_** | **-0.42^†§^ [-0.65 − -0.19]** | -0.12 [-0.36 – 0.12] | -0.21 [-0.52 – 0.07] | -0.02 [-0.23 – 0.25] |
|  | ***R_1_*** | -0.12 [-0.39 – 0.14] | 0.06 [-0.20 – 0.31] | 0.12 [-0.17 – 0.40] | 0.07 [-0.21 – 0.35] |
| **Lateral temporal** | **BP_ND_** | -0.21 [-0.47 – 0.04] | **-0.28*^§^ [-0.53 – -0.05]** | **-0.39^†§^ [-0.68** – **-0.14]** | -0.25 [-0.54 – 0.01] |
|  | ***R_1_*** | -0.04 [-0.29 – 0.21] | **0.25* [0.02 – 0.48]** | 0.27 [0.00 – 0.53] | **0.28* [0.03 – 0.53]** |
| **Parietal** | **BP_ND_** | -0.19 [-0.48 – 0.10] | **-0.49^‡§^ [-0.83 – -0.28]** | **-0.36*^§^ [-0.76 – -0.07]** | **-0.52^‡§^ [-0.89** – **-0.28]** |
|  | ***R_1_*** | 0.01 [-0.24 – 0.25] | **0.30^†§^ [0.08 – 0.52]** | 0.26 [-0.01 – 0.52] | **0.38^†§^ [0.13 – 0.60]** |
| **Occipital** | **BP_ND_** | -0.24 [-0.51 – 0.02] | **-0.28*^§^ [-0.55 – -0.04]** | **-0.42^†§^ [-0.75** – **-0.17]** | **-0.37^†§^ [-0.68 – -0.12]** |
|  | ***R_1_*** | 0.09 [-0.15 – 0.34] | 0.20 [-0.03 – 0.44] | 0.26 [-0.01 – 0.53] | **0.27* [0.03 – 0.54]** |
| **Frontal** | **BP_ND_** | -0.16 [-0.42 – 0.10] | **-0.38^†§^ [-0.69 – -0.18]** | -0.25 [-0.63 – 0.03] | **-0.34^†§^ [-0.68 – -0.10]** |
|  | ***R_1_*** | -0.23 [-0.47 – 0.02] | 0.11 [-0.13 – 0.35] | 0.11 [-0.17 – 0.38] | 0.10 [-0.16 – 0.37] |
| ***Model 2*** |  |  |  |  |  |
| **Medial temporal** | **BP_ND_** | -**0.43^‡§^ [-0.66** − **-0.20]** | -0.12 [-0.36 − 0.13] | -0.21 [-0.52 − 0.08] | -0.02 [-0.29 − 0.26] |
|  | ***R_1_*** | -0.16 [-0.40 − 0.09] | 0.05 [-0.20 − 0.30] | 0.11 [-0.17 − 0.38] | 0.07 [-0.21 − 0.35] |
| **Lateral temporal** | **BP_ND_** | -0.24 [-0.50 − 0.03] | -0.23 [-0.49 − 0.00] | **-0.35* [-0.64** − **-0.10]** | -0.20 [-0.48 − 0.07] |
|  | ***R_1_*** | -0.10 [-0.35 − 0.16] | 0.20 [-0.03 − 0.43] | 0.20 [-0.06 − 0.45] | 0.24 [-0.02 − 0.49] |
| **Parietal** | **BP_ND_** | -0.22 [-0.53 − 0.10] | -**0.42^†§^ [-0.76** − **-0.19]** | -0.30 [-0.71 − 0.00] | **-0.42^†§^ [-0.79** − **-0.16]** |
|  | ***R_1_*** | -0.06 [-0.31 − 0.20] | 0.18 [-0.03 − 0.40] | 0.18 [-0.09 − 0.45] | **0.26* [0.03** − **0.49]** |
| **Occipital** | **BP_ND_** | -0.25 [-0.54 − 0.05] | -0.23 [-0.52 − 0.04] | **-0.37*^§^ [-0.72** − **-0.10]** | **-0.30* [-0.62 − -0.02]** |
|  | ***R_1_*** | -0.00 [-0.27 − 0.26] | 0.12 [-0.13 − 0.37] | 0.13 [-0.15 − 0.41] | 0.16 [-0.10 − 0.44] |
| **Frontal** | **BP_ND_** | -0.17 [-0.42 − 0.08] | -**0.37^†§^ [-0.69** − **-0.18]** | -0.24 [-0.62 − 0.04] | **-0.33*^§^ [-0.68** − **-0.09]** |
|  | ***R_1_*** | -0.23 [-0.48 − 0.01] | 0.08 [-0.14 − 0.31] | 0.10 [-0.17 − 0.37] | 0.07 [-0.18 − 0.33] |

Models are adjusted for age, sex and education. Partial volume corrected [^18^F]flortaucipir BP_ND_ and *R_1_* were included in the model separately (model 1) and simultaneously (model 2). Standardized β’s with 95% confidence intervals are reported. BP_ND_ = non-displaceable binding potential. *p<0.05, ^†^p<0.01, ^‡^p<0.001, ^§^p_FDR_<0.05.
