# Supplementary figures and images for: Tau pathology and relative cerebral blood flow are independently associated with cognition in Alzheimer’s disease
Source: Eur J Nucl Med Mol Imaging. 2020 May 27;47(13):3165–75. doi: 10.1007/s00259-020-04831-w (PMC7680306; doi:10.1007/s00259-020-04831-w)

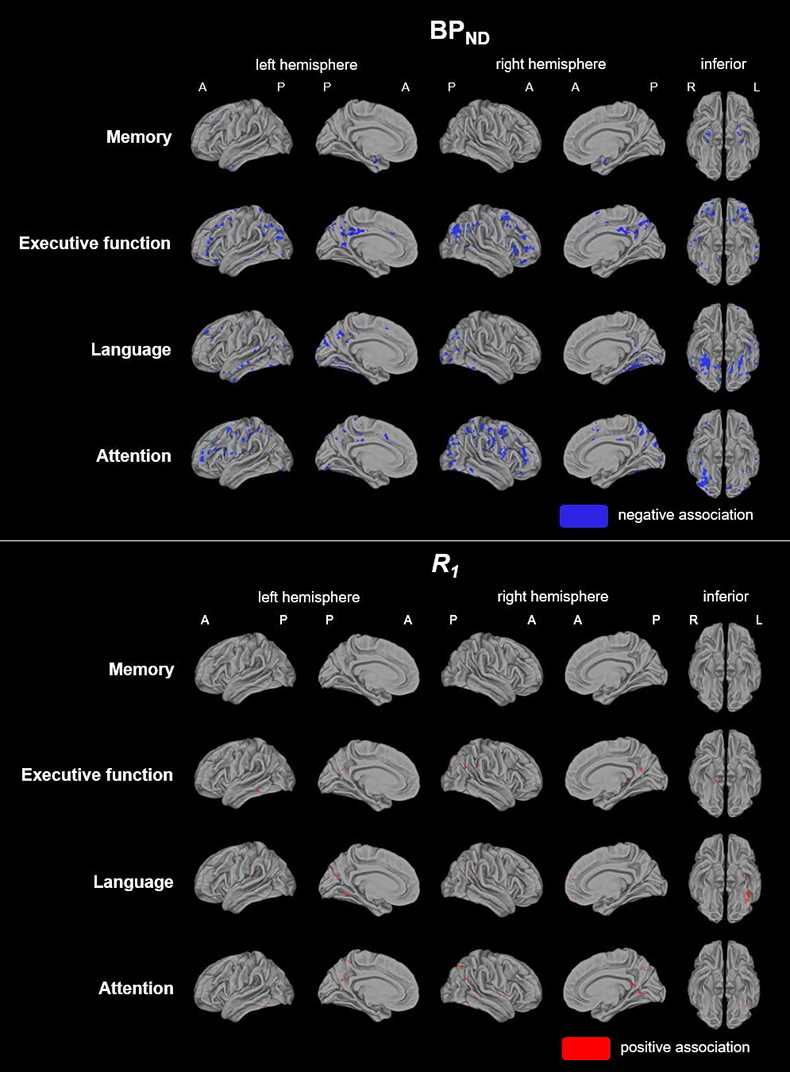

Supplement: Supplementary file 5 — (PNG 2485 kb). [file 259_2020_4831_Fig4_ESM.png]

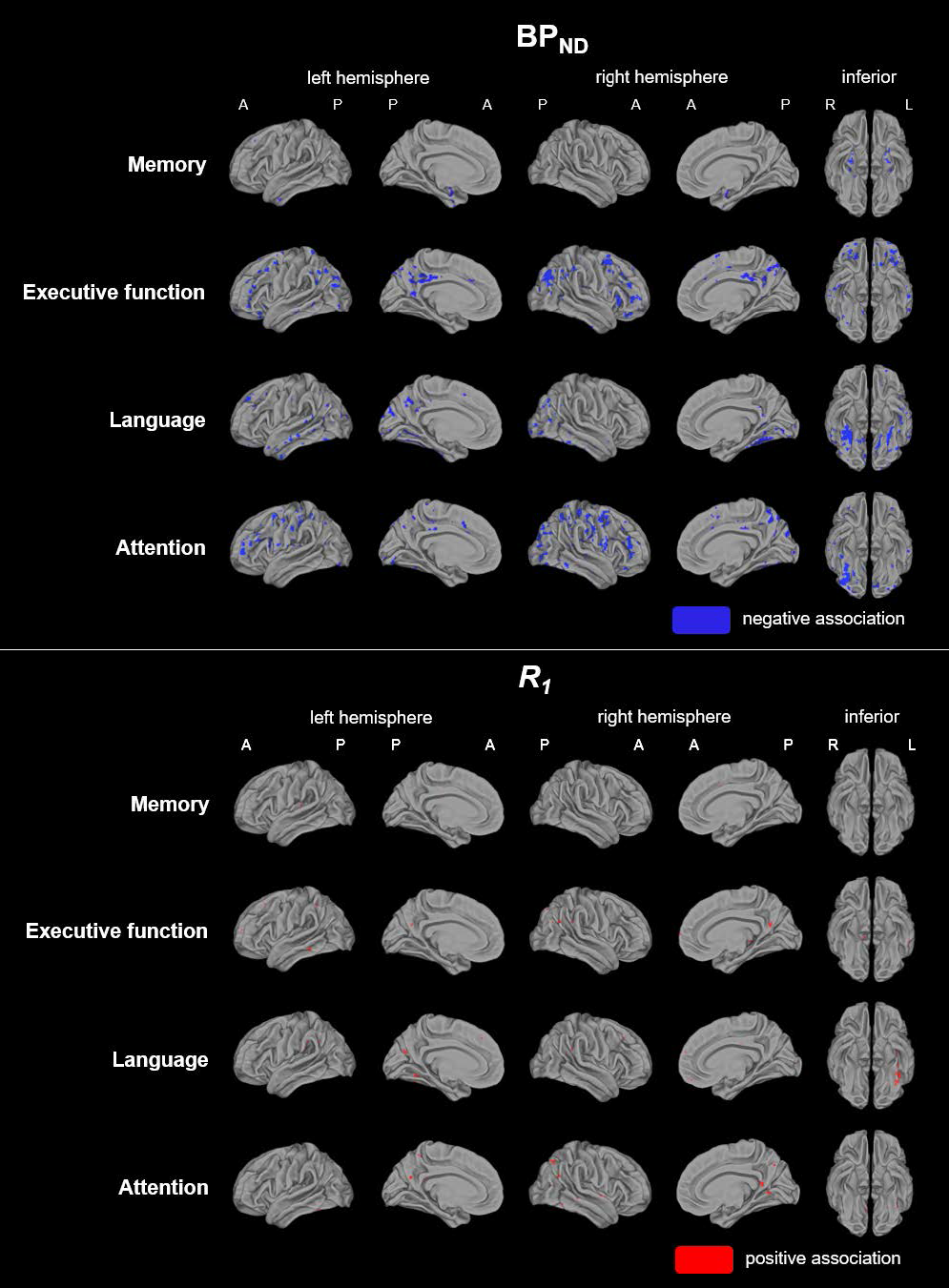

Supplement: Supplementary file 6 — High Resolution Image (TIFF 863 kb). [file 259_2020_4831_MOESM5_ESM.tiff]

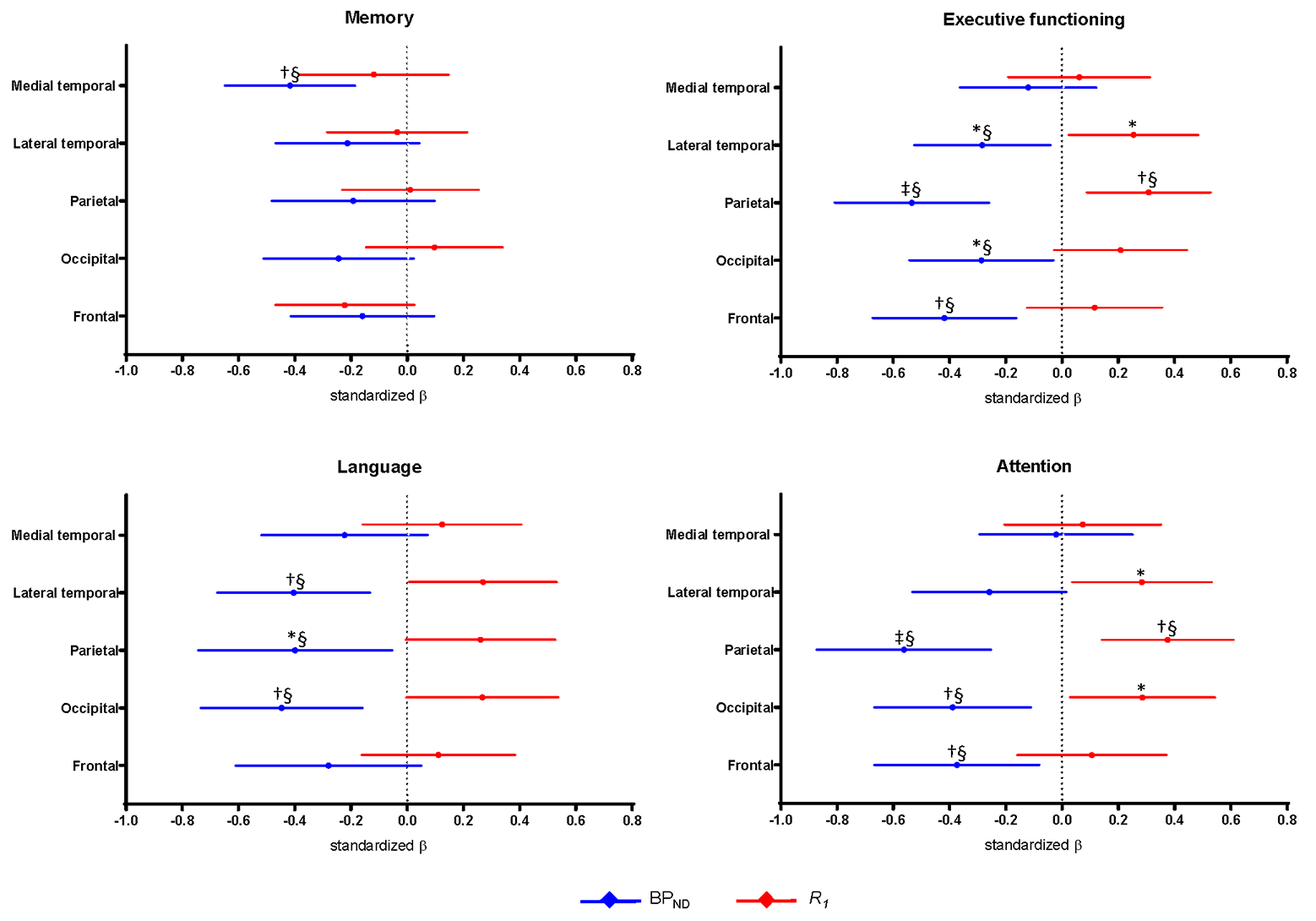

Supplement: Supplementary file 7 — (PNG 4979 kb). [file 259_2020_4831_Fig5_ESM.png]

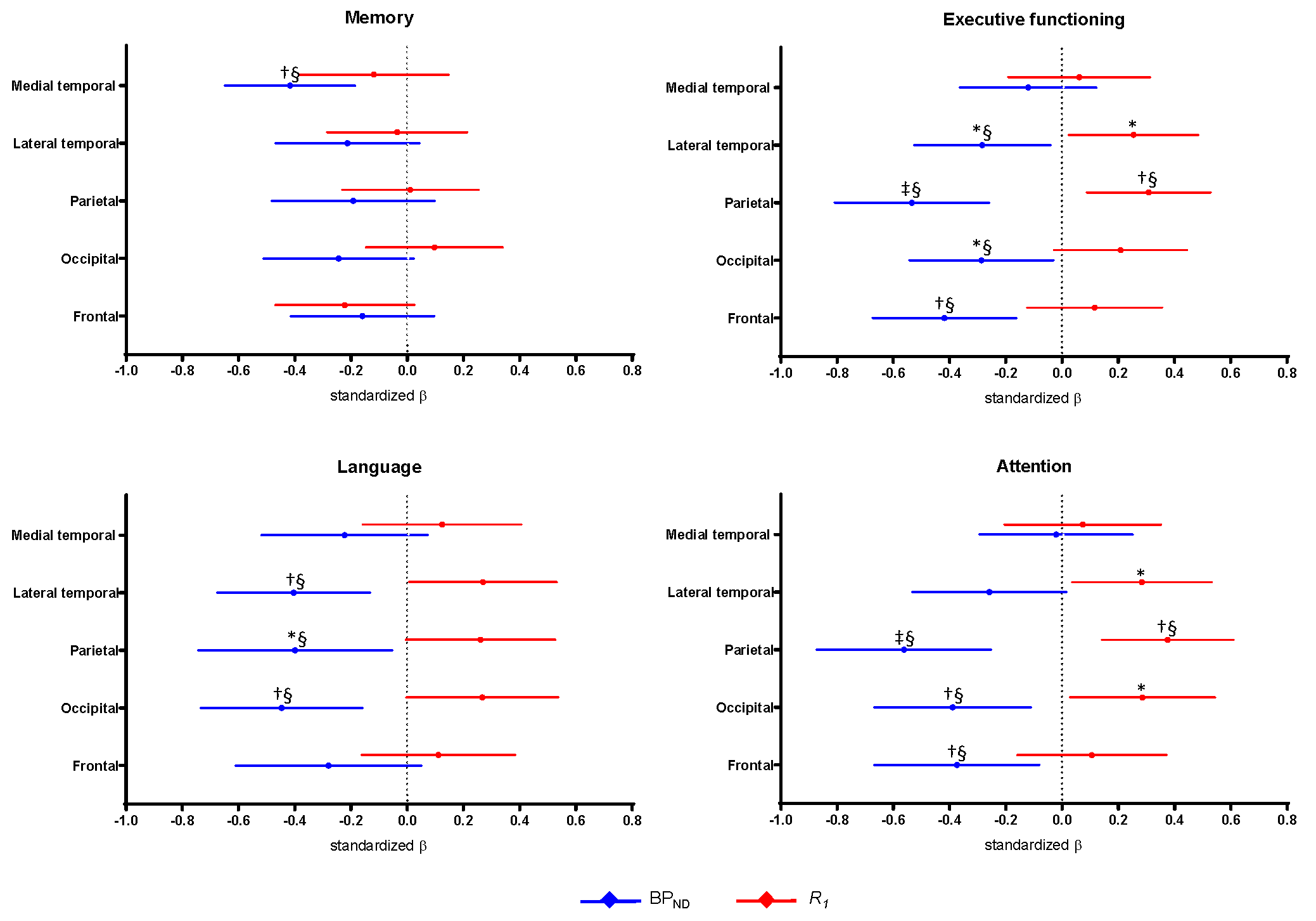

Supplement: Supplementary file 8 — High Resolution Image (TIFF 241 kb). [file 259_2020_4831_MOESM6_ESM.tiff]
